# A Glorious Distinction Flung Away for a Paltry Mess of Pottage—Behring’s Patent and Asserted Monopoly of Diphtheria Antitoxin

**Published:** 1898-09

**Authors:** 


### A Glorious Distinction Flung Away for a Paltry Mess of Pottage—Behring's Patent and Asserted Monopoly of Diphtheria Antitoxin.

As powerful and profound as is the tendency of war to demoralize the national character and to inflame the most brutal passions of individuals, it carries in its train of curses and sorrows at least one benefit of inestimable profit to society. War causes mankind to revise its standard of human values. In war we learn what we are too prone to forget in peace—that the proper measure of a man's worth and weight in human society is not his ability to accumulate money, not his power to outstrip his fellow men in the race for position or success, but, wholly and exclusively, his capacity for disinterested and generous service to his kind. All that is devoted, all the latent enthusiasms of the human breast, all the noble resources of self-sacrifice and fortitude, which glorify manhood, unite in times of war to declare that life is cheap but honor dear, and that the only career worth the following is that which leaves its mark for good on the community, the nation or race.

Every man who is not a confiding fool or a credulous dupe knows that these things are apt to be forgotten in the lazy, luxurious and selfish days of peace. Removed from the strenuous call of duty, danger and patriotism, the average man lapses into a mood that is fatal to every generous ideal of disinterested service to others; small, petty, self-seeking motives soon assert themselves; the demands of the higher life are habitually ignored; and the pursuits of the money-making and dollar-saving machine are resumed in utter forgetfulness of every purpose which can impart substance and validity to our lives.

From this censure should be justly exempted one class of men whose heroism in war is equalled only by their devotion in peace; who, at least in a large minority of cases, spend noble and laborious lives in combating disease, in ministering to the afflicted, in suppressing pestilence, in fronting with serene composure the hardships, dangers and fatigues of the medical practitioner. These men form the brightest ornaments of the human race, and in observing the marked contrast between their helpful lives and the besotted selfishness of many on whom they lavish an unrewarded toil, one cannot help wishing that there were some means of isolating and propagating the generous strain and then using it to enrich our deteriorated blood!

To their eternal praise be it further remembered that the choicer spirits of the medical profession have in all ages maintained and perpetuated a high and fine conception of the duty which the physician owes to his own dignity, to his calling, and to his pupils and to the community. They have realized keenly that disinterested, *unpaid*, *unbought* service on behalf of others is the only source of true distinction; that the real leaders of mankind are its unrequited servants; and that he who demands wages, profit, adequate pay for his work, must be content to sacrifice fame, reputation, influence. So, too, the man who has been paid for his brain or his blood remains forever the cheap mercenary. *He has been paid;* he has received his price; and he must not murmur when he is dismissed into the limbo of the hireling, unthanked, unhonored, unremembered!

It is this noble spirit which animates the code of medical ethics. Here is the only creed whose claims to professional salvation command recognition—a creed which enjoins both the manners of the gentleman and the generosity of the philanthropist—a creed which embodies the highest conception of professional dignity and honor—a creed which has been born of, and which has in turn begotten, all that is generous and worthy and inspiring in professional life—a creed which is alone capable of resisting the lowering, cheapening and degrading forces that threaten to transform Medicine into a trade.

Acting in the spirit of this creed, medical investigators have rarely felt justified in withholding from the great body of their colleagues the fruits of their researches. As they have partaken freely of the common fund of knowledge formed from the contributions of their predecessors, so they have felt constrained to enrich this common fund with their own legacy to posterity. A magnificent heritage has thus accumulated; and every land cherishes with pride, with gratitude and with affectionate veneration its bright muster-roll of departed sprits who gave without stint the best treasures and the choicest fruits of their toil.

Happily, this high tradition is yet a potent force amongst American practitioners, hence great will be the shock, profound the regret, when it is learned that no less a man than Emil Behring has sought to create for himself and his commercial agents a monopoly of diphtheria antitoxin; that not content with the fame and glory of his researches, or with the credit justly attaching to his share in establishing serum-therapy, he made application in January, 1895, for a United States patent on diphtheria antotoxin; that this patent was *five times refused* on the most cogent and substantial grounds, and in simple justice to the long line of bacteriological investigators who had clearly contributed to the results which Behring sought to monopolize; that after the lapse of three years, and after five distinct refusals, a patent was granted in June, 1898, by the Board of Appeals in Washington on the sole and avowed consideration that Behring's work had helped to reduce the diphtheria mortality; and that now the manufacturers of the Behring serum, his assignees known as the *Hochst Farbwerke*, have served notice of their monopoly on the leading American manufacturers, with menace of suit if the German monopoly be not respected.

On the behavior of the *Farbwerke* (*vormals Meister*, *Lucius & Bruning*) we have no comment to make: they have acted in accordance with their lights. They are a commercial house and pretend to no higher motives than those of the average business man. Patents on medicinal substances, monopolies, exploitation of the American public to the full extent permitted by our criminally indulgent laws—these things are mere matters of business in eyes so long accustomed to that ignoble scramble for patents and monopolies, which stains and besmirches the recent annals of German science. The Germans affect to despise the sordid motives and the frantic dollar-chase of the Americans; "Americanismus" is a German term of reproach; but in no land and in no degenerate age have scientific men been more prone than today in Germany to throw professional dignity and professional duty to the winds, joining in the wild pursuit of monopolies and dollars as frankly and scynically as any American "promoter." If we Americans have been the teachers, the Germans proved apt pupils; and they are now abundantly qualified to instruct their preceptors.

But from this whole disgusting and paltry rapacity a few German scientists had thus far held aloof. Rudolph Virchow is one of them. Emil Behring *was* another. Until recently Behring belonged to that noble band of chosen spirits whose names were handed down from generation to generation as the benefactors of mankind. What is he today? With sorrow we answer: the cheap and mercenary monopolist who tarnishes for money the luster of his scientific fame; the fit companion of those who originated antipyrin, phenacetine, salol—*and received their price;* the Esau of German science who flings away for a beggarly mess of pottage his claim to a glorious immortality, and an ethical standing which, though it cannot be weighed or gauged, or expressed in dollars and cents, should be as precious to its possessor as a woman's chasity or a man's honor; nay, more—and mark this well—the greedy and unscrupulous appropriator of other men's researches; the spurious claimant of reward for work done in large measure by Pasteur, Roux, Sewell, Fraenkel, Foa, Bonome, Kitasato, Wernicke, Aronson, Haricourt, Richet, Emmerich, Ogata, Jasuhara, Tizzoni, Cattani, Ehrlich and many others.*

But though Professor Behring has seen fit to descend to the level of monopolists and promoters, he has reckoned without his host and he may yet vainly seek his reward. Not a court in the United States will uphold such a preposterous patent; the claims of his minions will be disputed and fought to the last trench; and in the sequal he may find that there is a limit both to the exploitation of American patent laws, and the appropriation of credit for other men's work.

Meantime, be it remembered to our shame as Americans, that in Germany the very claim for such a patent would be scouted and repelled. The laws of Germany and France withhold all patents on foods and medicines, save on processes of manufacture, and in this instance Behring could not possibly secure at home what he has been granted in the United States. In Germany he accepts the situation and makes the best of the existing competition; in America he would suppress all competition and remain undisputed master of the field! Is it not high time to bring some organized effort to bear on Congress for a change in the patent laws? Is it not a scandal and a shame that foreigners should enjoy in America monopolies and concessions which are denied them at home? Consider the oppressive extortion to which this has given rise. Remember phenacetine, sulphonal, antipyrin, salol—marketed here at prices outrageously excessive. Remember that not an ounce of these products may be legally purchased in Canada and imported into the United States *duty paid.* Is there no limit to American patience? How long shall we continue to tolerate

*See chapter on "Acquired Immunity," Sternberg's "Immunity and Serum Therapy."

the foreigner's extortion—how long will he fatten on the monopolies which American laws create for him?—From Advanced Sheets of Editorials in August, '98, *Bulletin of Pharmacy*, Detroit.

